# Higher educational attainment in Huntington disease families: evidence from the Enroll-HD study

**DOI:** 10.1186/s13023-026-04336-z

**Published:** 2026-04-09

**Authors:** Jesus E. Vazquez, Dewei Lin, Adys Mendizabal, Amy C. Ogilvie, Elizabeth A. Stuart, Tanya P. Garcia

**Affiliations:** 1 Department of Biostatistics, Bloomberg School of Public Health, Johns Hopkins University, Baltimore, MD USA; 2 Department of Biostatistics, Gillings School of Global Public Health, The University of North Carolina at Chapel Hill, Chapel Hill, NC USA; 3 Department of Neurology, University of California, Los Angeles, CA USA; 4 Health Services Research Division, Department of Neurology, Ohio State University, Columbus, OH USA

**Keywords:** Huntington disease (HD), Educational attainment, Social determinants, Health inequities, Rare diseases, Life-course epidemiology

## Abstract

**Background:**

Huntington disease (HD) is an inherited neurodegenerative disorder that impairs motor, cognitive, and psychiatric function. Offspring of individuals with HD may experience early caregiving responsibilities, potentially disrupting their educational outcomes. We evaluated the associations between parental age at HD symptom onset, genetic-expansion, sociodemographic, and regional factors with offspring educational attainment outcomes in adulthood.

**Methods:**

We estimated odds ratios using logistic regression to evaluate associations between higher educational attainment in offspring and parental age at symptom onset, genetic-expansion, race, and region among adults ($\:\ge\:$18 years) in the Enroll-HD study. To assess the relative importance of exposures in predicting educational outcomes, we fit a random forest model and ranked these based on mean decrease in accuracy.

**Results:**

In our explorative analysis, participants whose parents had an earlier age at HD symptom onset were associated with lower odds of attaining a higher education. We also identified a nonlinear, inverted-U association between genetic-expansion and the probability of higher educational attainment–a pattern that has been observed in prior studies of neurocognitive function in children. Marked differences were also observed by race and region: Black, Hispanic/Latino, and Native American participants were associated with lower odds of higher education compared with White participants, and those residing outside Northern America were associated with lower odds of higher educational attainment.

**Discussion:**

Earlier parental HD onset was associated with lower educational attainment in offspring and disparities were observed across genetic-expansion, sociodemographic, and regional groups. Our exploratory findings may inform future studies aimed at better understanding educational inequities among families affected by HD and related neurodegenerative disorders.

**Supplementary Information:**

The online version contains supplementary material available at 10.1186/s13023-026-04336-z.

## Background

Huntington disease (HD) is an autosomal dominant, fully penetrant neurodegenerative disorder that progressively impairs motor, cognitive, and psychiatric functioning [1]. It is predominantly characterized by involuntary movements (i.e., chorea), although cognitive and psychiatric symptoms may predate onset of motor symptoms by one to two decades [2]. The disorder is caused by an abnormal expansion of a cytosine-adenine-guanine (CAG) trinucleotide repeat in the HTT gene on chromosome 4, with individuals with 40 CAG repeats or longer experiencing a typical onset of symptoms between 30 and 50 years of age, with longer CAG repeats associated with earlier onset of symptoms and more severe disease symptomatology [2, 3]. Most individuals die due to HD and disease-related complications within one to two decades following the onset of motor symptoms [3].

HD remains a rare disease, but its public-health impact is expanding: recent estimates suggest that 13–15 per 100,000 individuals in the United States (US) are affected, and age-adjusted mortality increased from 4.3 to 6.0 per 100,000 between 1999 and 2020 [4, 5]. HD also exhibits genetic anticipation, whereby the mutation expands across generations–especially when inherited from the father–resulting in earlier onset and more severe disease in offspring [6]. In the absence of disease-modifying therapies [7], the combination of rising mortality and intergenerational anticipation underscores the importance of identifying life-modifying factors that improve long-term wellbeing among individuals with HD and their families.

Specifically, children growing up in families affected by HD often face overlapping challenges. When a parent develops symptoms during a child’s formative years, the emotional and financial toll of caregiving can disrupt family structures [8, 9]. Children may assume caregiving roles while coping with uncertainty about their own genetic risk, leading to heightened stress, reduced educational opportunities, and limited social mobility [10]. Yet how the timing of parental symptom onset affects educational outcomes remains unclear. Understanding these relationships is critical for designing support systems that mitigate the potential lower educational consequences of early parental onset.

Higher educational attainment is an important life-modifiable factor as it supports cognitive reserve–the brain’s ability to withstand neurodegeneration [11]–fosters health literacy and increases access to material and social resources [12]. Greater cognitive reserve has been associated with better social engagement and overall wellbeing in later life [13]. In HD, higher educational attainment has been linked to slower disease progression, better functional mobility, and improved short- and long-term social inclusion and participation [14, 15]. Beyond HD, higher educational attainment is associated with lower risks of depression, cardiovascular disease, and cognitive decline, each of which contributes to overall brain health and long-term wellbeing [16–19]. Early disruptions to education–such as those caused by parental illness–can limit educational attainment and contribute to widening health inequities over the life course.

Qualitative studies have documented early disruptions in HD families. Caregivers frequently worry about children’s wellbeing, including disrupted parent-child relationships, financial insecurity, and the children’s own risk of developing HD [10]. The financial insecurity can compound these stresses as family resources are redirected toward caregiving or medical costs–and when the parent is the main wage earner, loss of income further limits the support for children’s educational development. Moreover, individuals raised in HD families report elevated levels of childhood trauma and psychological distress in adulthood, reflecting the long-term emotional impact of growing up in an environment shaped by hereditary illness and caregiving demands [20].

Despite the mounting qualitative evidence of the importance of education as a modifiable determinant of wellbeing and HD progression, research has primarily treated education as a factor explaining clinical outcomes, rather than investigating the factors that shape educational attainment itself [7, 15]. Existing work has relied on qualitative studies, leaving limited empirical evidence on how parental onset, genetic-expansion, sociodemographic, and regional factors shape educational outcomes. Furthermore, there is limited reporting on the role of other social and structural determinants of health, including race, country of residence and its interactions with educational attainment and clinical outcomes in HD.

Early parental symptom onset may be associated with disruptions in family stability, increase caregiving demands, and limit educational opportunities. At the genetic level, higher CAG repeat lengths generally lead to worse health outcomes [7]; however, higher repeat counts have also been associated with advantageous neurodevelopment and cognitive performance in children [21], though whether these cognitive advantages translate to educational outcomes remains unknown. Racial and ethnic disparities in high school completion are well documented in the US population [22, 23], yet HD research has historically underrepresented diverse populations, limiting the understanding of how such disparities manifest within HD families. Finally, regional differences in educational attainment exist between the US and European Union (EU) [24, 25], but whether these patterns persist in the HD community remains unknown.

To address these gaps, we used data from the Enroll-HD study to evaluate whether a later parental age at symptom onset is associated with higher educational attainment, whether CAG repeat length shows a nonlinear relationship with educational outcomes, and whether these patterns differ across sociodemographic and regional subgroups. Understanding how these factors influence educational attainment in offspring can inform strategies that promote educational equity in affected HD families.

## Methods

### Study design and data source

We used data from the Enroll-HD study (Periodic Dataset 5; 2012–2020), an ongoing observational study of HD. Enroll-HD includes participants who have been diagnosed with HD, participants at risk based on family history, as well as family members without HD and community controls. Data were collected across 61 clinical sites in Australasia, Europe, Latin America, and North America. All participants provided written informed consent, directly or through a legally authorized representative, and the study was approved by the local ethics committees of participating sites. The Enroll-HD consent permits the use of de-identified data in secondary research analyses, for which the present study used, and was conducted in accordance with ethical regulations. A detailed description of study protocol can be found elsewhere [26, 27].

### Analytic sample selection

**Fig. 1:**
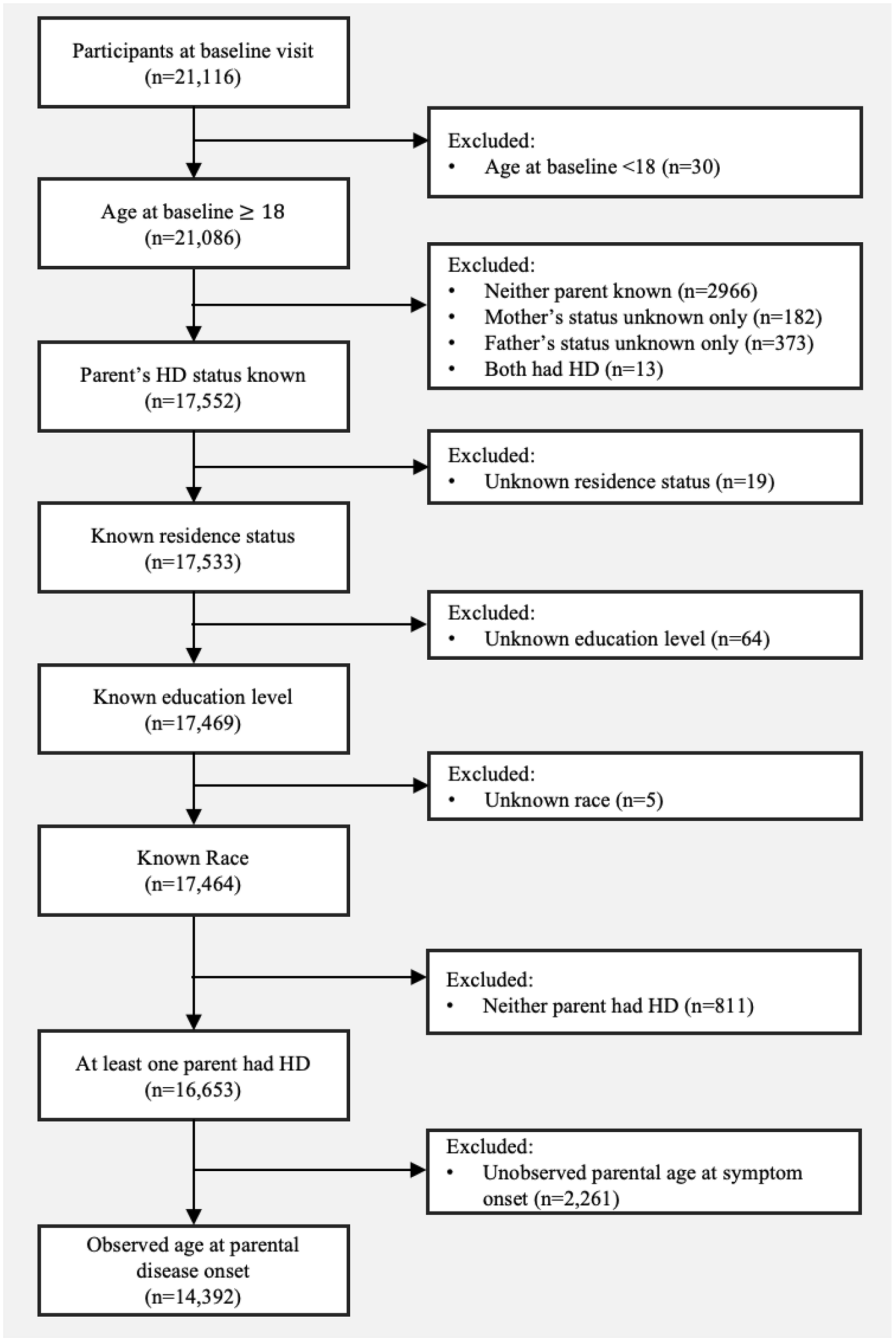
Analytical sample selection of Enroll-HD study (*n* = 14,392)

We restricted the analytical sample to participants who were $\:\ge\:$18 years of age at baseline (*n* = 21,086). Participants were eligible for inclusion regardless of whether they themselves had developed HD related symptoms or received a diagnosis; our aim was to evaluate educational attainment across the full range of participants whose parent(s) had HD. Then, we applied sequential exclusion criteria (Fig. 1): participants without documented parental HD status (*n* = 3,534 excluded), those with both parents affected by HD (*n* = 13 excluded), those with missing covariate data (*n* = 75 excluded), and those who reported that neither parent had HD (*n*=811 excluded). Finally, we restricted the sample to participants with observed parental age at symptom onset, excluding those with right-censored data (parent had not yet exhibited symptoms at time of interview), missing data, or invalid entries (*n* = 2,261 excluded). The final analytical sample consisted of *n* = 14,392 participants (offspring) for whom parental age at disease onset–reflecting the parent’s age when they first presented symptoms–was observed.

### Measures

#### Educational attainment

The primary outcome, higher educational attainment, was defined as an International Standard Classification of Education (ISCED, 2011 version) level $\:\ge\:3$, corresponding to completion of at least an upper-secondary education. Educational attainment was operationalized as a structural life-course indicator rather than a direct measure of social functioning. While the ISCED was designed to facilitate cross-national comparisons, educational systems are heterogeneous, and dichotomizing ISCED levels may obscure differences.

#### Parental age at symptom onset

Parental age at onset, the main exposure of interest, was collected by asking participants to report the age (years) at which their parent first presented HD symptoms. To improve the interpretability of the results we operationalized it both as a continuous and categorized variable: <25, 25–34, 35–44, and $\:\ge\:$45 years. Symptoms may have been motor, cognitive, or/and psychiatric, but information related to symptom type is unknown. Additionally, the parental age at symptom onset may not coincide with the age at which the parent first received a motor diagnosis, which is defined by a diagnostic confidence level of 4 (DCL4) on the 1999 Unified Huntington’s Disease Rating Scale [27, 28]. Thus, the measure reflects the participant’s perception of their parent’s earliest symptomatic changes rather than a formal motor clinical diagnosis.

#### Genetic and sociodemographic measures

Given the importance of genetic-expansion on disease severity, we included and operationalized CAG repeat length both as a continuous and categorized variable: normal (< 27), intermediate (27-35), reduced penetrance (36–39), full-penetrance (40–59), and high ($\:\ge\:$60) [29, 30]. We additionally investigated self-reported race (White, Black, Hispanic/Latino, Asian, Native American, or Other) and region (Australasia, Europe, Latin America, Northern America); factors that may independently impact educational attainment based on social and structural determinants of health. Additional sociodemographic exposures included participant self-reported sex recorded based on a single baseline item with two response options (male, female), and affected parent (father, mother).

Participants entered the study at different ages, raising the possibility that some participants may have a differential advantage in completing their education at enrollment. Therefore, we adjusted for age at baseline (years) to address age-related differences in educational opportunities and generational shifts in educational access. Moreover, participants may also have gained access to additional resources after enrollment; therefore, all analyses were restricted to baseline rather than last-visit data to minimize potential bias introduced by study participation. Residence (village, town, city, or rural) was additionally considered as a sociodemographic factor; because residential location may have changed after educational completion, this covariate was included only for sensitivity analyses.

### Statistical analyses

A logistic regression model was used to estimate the odds of higher educational attainment as a function of parental age at onset, CAG repeat length, sex, race, region, baseline age, and affected parent; regression estimates were adjusted for all listed covariates. Parental age at onset and CAG repeat length were modeled categorically to estimate the odds ratio (OR) of higher educational attainment. In a secondary analysis, parental age at onset and CAG repeat length were modeled using restricted cubic splines to allow for nonlinearity and mitigate high variability arising from sparse data in certain age ranges. The Hosmer-Lemeshow goodness of fit test was used to assess model fit. A sensitivity analysis included residence as a covariate and excluded cases of juvenile HD–defined as reaching a DCL4 before the age 20 [30]–as these participants may follow distinct symptomology and educational trajectories. Given the small counts in some race and regional subgroups, estimates should be interpreted with caution.

To assess the relative predictive importance of covariates identified in the logistic regression model, we fit a random forest classification model. The random forest was used as a complementary, non-parametric approach rather than as an additional analysis. Model tuning was based on the Kappa statistic–which adjusts for agreement due to chance–using a 5-fold cross-validation approach. The best model consisted of an ensemble of 1,000 decision trees and achieved the best performance when all predictor variables were considered. Predictive accuracy was summarized using the area under the receiver operating characteristic curve (AUC). Variable importance was measured using the Mean Decrease in Accuracy (MDA) was operationalized as high (MDA > 25), moderate (10$\:\le\:$MDA$\:\le\:$25), or low (MDA<10).

We conducted univariate analyses to examine associations between higher educational attainment and each variable. For categorical variables, we use chi-square tests; for continuous variables, we used Kruskal-Wallis rank-sum tests to compare medians across groups. Descriptive statistics included medians and interquartile ranges (IQRs) for continuous variables and frequencies with percentages for categorical variables, reported overall and stratified by educational attainment status. Groups (and surrounding groups) with counts in the range 1–5 were suppressed to mitigate risk of identifiability. Wald-type 95% confidence intervals (CIs) were computed for all regression estimates, and statistical significance was assessed at the $\:{\upalpha\:}$=0.05 level. All analyses were conducted using R version 4.4.2.

## Results

### Study characteristics

Overall, 84% (*n* = 12,061) reported a higher educational attainment. Most participants were female (57%), from Europe (62%), and White (94%) (Table 1). When stratified by educational attainment, marked differences emerged by sex, region, residence, race, age at baseline, CAG repeat length, and parental age at onset. Participants with higher educational attainment, compared to those with lower attainment, were more likely to have a parent whose age at onset was $\:\ge\:$45 years (63% vs. 60%; *p* = 0.02) and have a normal CAG repeat length (14% vs. 9%; *p* < 0.001). Similarly, when stratified by parental age at onset, marked differences appeared across region, race, age at baseline, CAG repeat length, and affected parent. The mother was the most commonly affected parent overall and within each parental age at onset group; however, this proportion decreased as parental age at onset increased–from 71% for onset <25, to 62% for ages 25–34, 56% for ages 35–44, and 53% for onset ≥45.

**Table 1:** Analytical sample characteristics, Enroll-HD (*n* = 14,392)

Percentages % (*n*)	Overall (*n* = 14,392)	Higher educational attainment		*P*-value^b^	Parental age of onset				
		**No (*n* = 2**,**331)**	**Yes (*n* = 12**,**061)**		$\:<25$ **(** ***n*** ** = 187)**	$\:25-34$**(*n* = 1**,**334)**	$\:35-44$**(*n* = 3**,**833)**	$\:\ge\:45$**(*n* = 9**,**038)**	*P*-value^b^
**Offspring characteristics**									
**Higher educational attainment**		-		-					0.016
Yes	83.8% (12,061)	-	-		80.7% (151)	81.8% (1,091)	83.0% (3,182)	84.5% (7,637)	
No	16.2% (2,331)	-	-		19.3% (36)	18.2% (243)	17.0% (651)	15.5% (1,401)	
**Sex**									0.160
Female	56.8% (8,172)	54.3% (1,266)	57.3% (6,906)		57.2% (107)	59.2% (790)	57.3% (2,198)	56.2% (5,077)	
Male	43.2% (6,220)	45.7% (1,065)	42.7% (5,155)		42.8% (80)	40.8% (544)	42.7% (1,635)	43.8% (3,961)	
**Region**				< 0.001					< 0.001
Northern America	33.6% (4,842)	4.9% (114)	39.2% (4,728)		47.6% (89)	37.7% (503)	34.2% (1,310)	32.5% (2,940)	
Europe	61.7% (8,875)	92.1% (2,147)	55.8% (6,728)		**	58.1% (775)	61.6% (2,361)	**	
Australasia	3.6% (525)	1.8% (42)	4.0% (483)		**	2.9% (39)	3.1% (119)	**	
Latin America	1.0% (150)	1.2% (28)	1.0% (122)		**	1.3% (17)	1.1% (43)	**	
**Residence**				< 0.001					0.200
Rural	5.9% (854)	5.4% (127)	6.0% (727)		7.5% (14)	5.2% (70)	5.7% (217)	6.1% (553)	
Village	16.3% (2,344)	22.5% (524)	15.1% (1,820)		14.4% (27)	13.9% (185)	16.4% (627)	16.7% (1,505)	
Town	35.0% (5,038)	41.6% (969)	33.7% (4,069)		38.5% (72)	36.0% (480)	35.2% (1,350)	34.7% (3,136)	
City	42.8% (6,156)	30.5% (711)	45.1% (5,445)		39.6% (74)	44.9% (599)	42.8% (1,639)	42.5% (3,844)	
**Race**				< 0.001					< 0.001
White	93.5% (13,457)	95.4% (2,223)	93.1% (11,234)		89.3% (167)	91.7% (1,223)	92.9% (3,561)	94.1% (8,506)	
Black	0.7% (103)	0.3% (6)	0.8% (97)		**	1.0% (14)	1.0% (39)	**	
Hispanic/Latino	2.2% (320)	1.9% (45)	2.3% (275)		**	2.3% (31)	2.6% (101)	**	
Native American	0.5% (65)	1.1% (26)	0.3% (39)		**	0.7% (10)	0.4% (17)	**	
Asian	0.7% (97)	0.7% (16)	0.7% (81)		**	1.0% (14)	0.8% (29)	**	
Other	2.4% (350)	0.6% (15)	2.8% (335)		4.8% (9)	3.1% (42)	2.2% (86)	2.4% (213)	
**Age at baseline, median (IQR)**	46.0 (35.0–56.0)	49.0 (39.0–59.0)	45.0 (35.0–55.0)	< 0.001	32.0 (25.5–39.0)	35.0 (28.0–43.0)	41.0 (32.0–50.0)	50.0 (40.0–59.0)	< 0.001
**CAG repeat length, median (IQR)**	42.0 (40.0–45.0)	43.0 (41.0–45.0)	42.0 (40.0–44.0)	< 0.001	48.0 (43.0–53.0)	46.0 (43.0–50.0)	44.0 (42.0–46.0)	42.0 (40.0–43.0)	< 0.001
**CAG repeat length group**				< 0.001					< 0.001
Normal ($\:<27$)	13.7% (1,965)	9.1% (213)	14.5% (1,752)		12.8% (24)	13.0% (173)	13.6% (521)	13.8% (1,247)	
Intermediate ($\:27-35$)	1.0% (142)	0.7% (17)	1.0% (125)		**	1.3% (17)	0.9% (34)	**	
Reduced penetrance ($\:36-39$)	3.8% (552)	3.5% (82)	3.9% (470)		**	0.6% (8)	1.3% (48)	**	
Full penetrance ($\:40-59$)	80.8% (11,635)	85.1% (1,984)	80.0% (9,651)		73.3% (137)	81.7% (1,090)	83.6% (3,206)	79.7% (7,202)	
High ($\:\ge\:60$)	0.7% (98)	1.5% (35)	0.5% (63)		**	3.4% (46)	0.6% (24)	**	
**Parental characteristics**									
**Parental age at onset, median (IQR)**	48.0 (40.0–56.0)	48.0 (40.0–56.0)	48.0 (40.0–56.0)	0.201	20.0 (19.5–23.0)	30.0 (30.0–32.0)	40.0 (37.0–40.0)	55.0 (50.0–60.0)	-
**Parental age at onset group**				0.016					-
$\:\:\:<25$	1.3% (187)	1.5% (36)	1.3% (151)		**-**	**-**	**-**	**-**	
$\:\:\:25-34$	9.3% (1,334)	10.4% (243)	9.0% (1,091)		**-**	**-**	**-**	**-**	
$\:\:\:35-44$	26.6% (3,833)	27.9% (651)	26.4% (3,182)		**-**	**-**	**-**	**-**	
$\:\:\:\ge\:45$	63.0% (9,038)	60.1% (1,401)	63.3% (7,637)		**-**	**-**	**-**	**-**	
**Affected parent**				0.750					< 0.001
Mother	54.6% (7,858)	54.9% (1,280)	54.5% (6,578)		70.6% (132)	62.2% (830)	56.1% (2,151)	52.5% (4,745)	
Father	45.4% (6,534)	45.1% (1,051)	45.5% (5,483)		29.4% (55)	37.8% (504)	43.9% (1,682)	47.5% (4,293)	

Note: Abbreviations: CAG (Cytosine-Adenine-Guanine), HD (Huntington disease), IQR (Interquartile range:$$\:{25}^{\mathrm{th}}-{75}^{\mathrm{th}}$$percentiles).$$\:{\boldsymbol{}}^{\boldsymbol{a}}$$Higher educational attainment defined as having an International Standard Classification of Education level$$\:\ge\:3$$$$\:{\boldsymbol{}}^{\boldsymbol{b}}$$For categorical variables,$$\:\boldsymbol{p}$$-values are from chi-square tests. For continuous variables,$$\:\boldsymbol{p}$$-values were obtained using the Kruskal-Wallis rank-sum test. **Groups (and surrounding groups) with counts in the range 1–5 were suppressed to mitigate risk of identifiability

### Regression analyses

#### Parental age at symptom onset

**Fig. 2:**
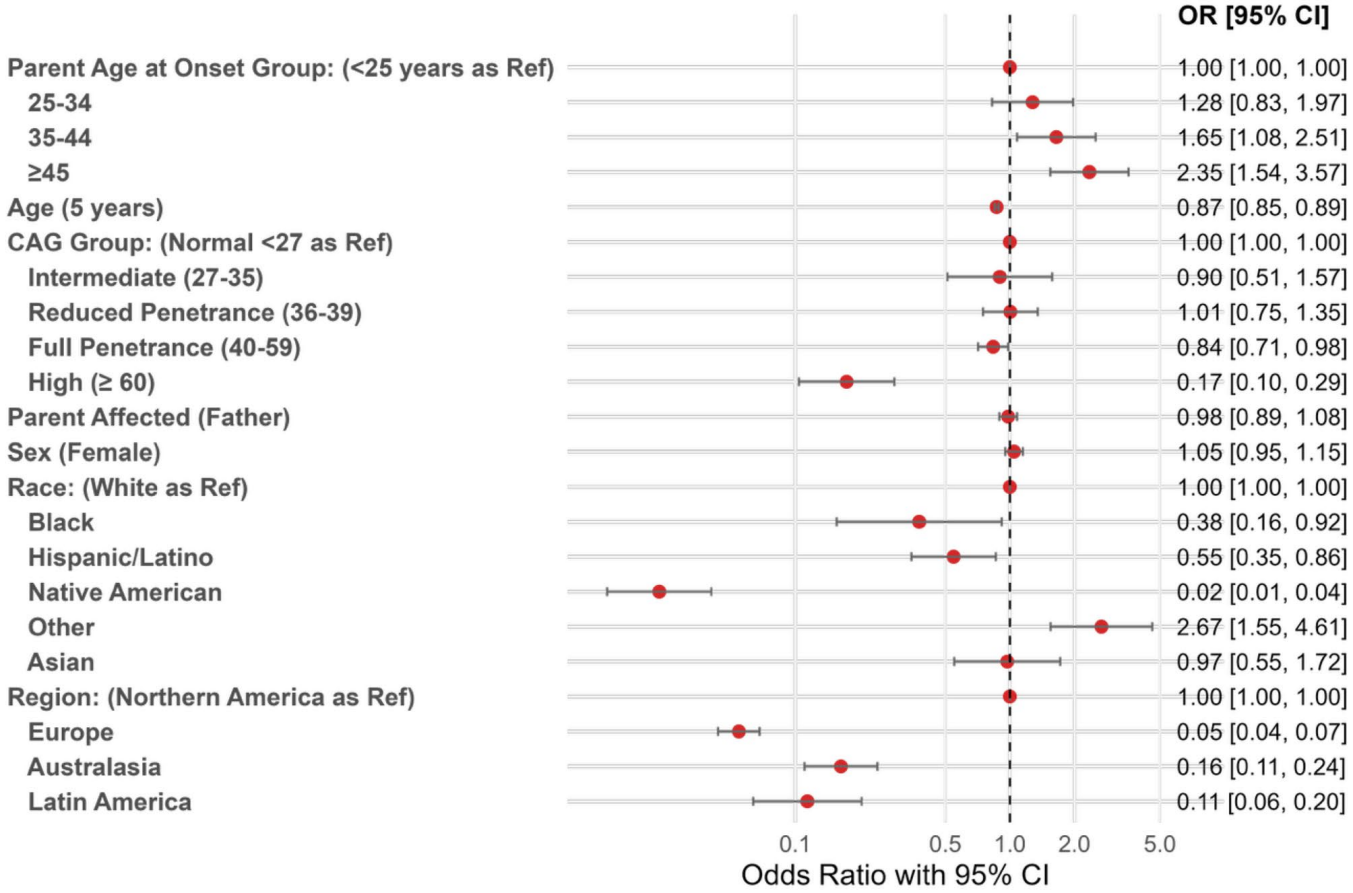
Odds ratios for higher educational attainment, Enroll-HD disease (*n* = 14,392). Legend: Odds ratio (OR) estimates and 95% confidence intervals of higher educational attainment. Estimates shown correspond to those provided in Table A1

When parental age at onset was modeled categorically, participants whose parent’s symptom onset occurred at age ≥ 45 years had significantly higher odds of higher educational attainment (OR = 2.35, 95% CI: 1.54–3.57) compared with those whose parent’s symptoms began before age 25 (Fig. 2, Table A1). Similarly, while not significant, parental onset between ages 35–44 and 25–34 were similarly associated with increased odds of higher educational attainment. These findings suggest that a later parental age at symptom onset was, on average, associated with higher educational attainment outcomes in offspring. Because educational systems differ across regions, we additionally conducted a stratified analysis by region. The overall pattern of higher educational attainment with later parental onset was broadly consistent across regions (Table A2).

#### Genetic and sociodemographic measures

Participants with intermediate (OR = 0.90; 95% CI: 0.51–1.57), full-penetrance (OR = 0.84; 95% CI: 0.71–0.98), or high (OR = 0.17; 95% CI: 0.10–0.29) CAG repeat length were found associated with lower odds of higher educational attainment relative to those with normal repeat lengths, whereas those with reduced penetrance showed slightly higher odds (OR = 1.01; 95% CI: 0.75–1.35) (Fig. 2). While only marked differences were observed for the full-penetrance and high repeat-length groups, the overall pattern across CAG repeat length categories suggested a nonlinear relationship between genetic-expansion and educational attainment outcomes. More specifically, the association appeared non-monotonic across repeat length categories–an association that was further observed in the restricted cubic spline analyses.

Differences in educational attainment were observed across race and regional groups as well. Relative to White participants, lower odds of higher educational attainment were observed among Black (OR = 0.38; 95% CI: 0.16–0.92), Hispanic/Latino (OR = 0.55; 95% CI: 0.35–0.86), and Native American (OR = 0.02; 95% CI: 0.01–0.04) participants. Additionally, relative to participants residing in Northern America, lower odds of higher educational attainment were observed among participants residing in Europe (OR = 0.06; 95% CI: 0.04–0.07), Australasia (OR = 0.16; 95% CI: 0.11–0.24), and Latin America (OR = 0.11; 95% CI: 0.06–0.20). These results must be interpreted with caution given the small counts for Black (*n* = 103), Hispanic/Latino (*n* = 320), and Native American (*n* = 65), and Latin America (*n* = 150) participants. Moreover, the aggregation of heterogeneous countries within regions may have also masked important regional differences. We report differences across racial and regional groups to improve representation in HD research. Still, the estimates should be viewed as descriptive rather than precise population-level effects.

#### Restricted cubic spline analyses

**Fig. 3:**
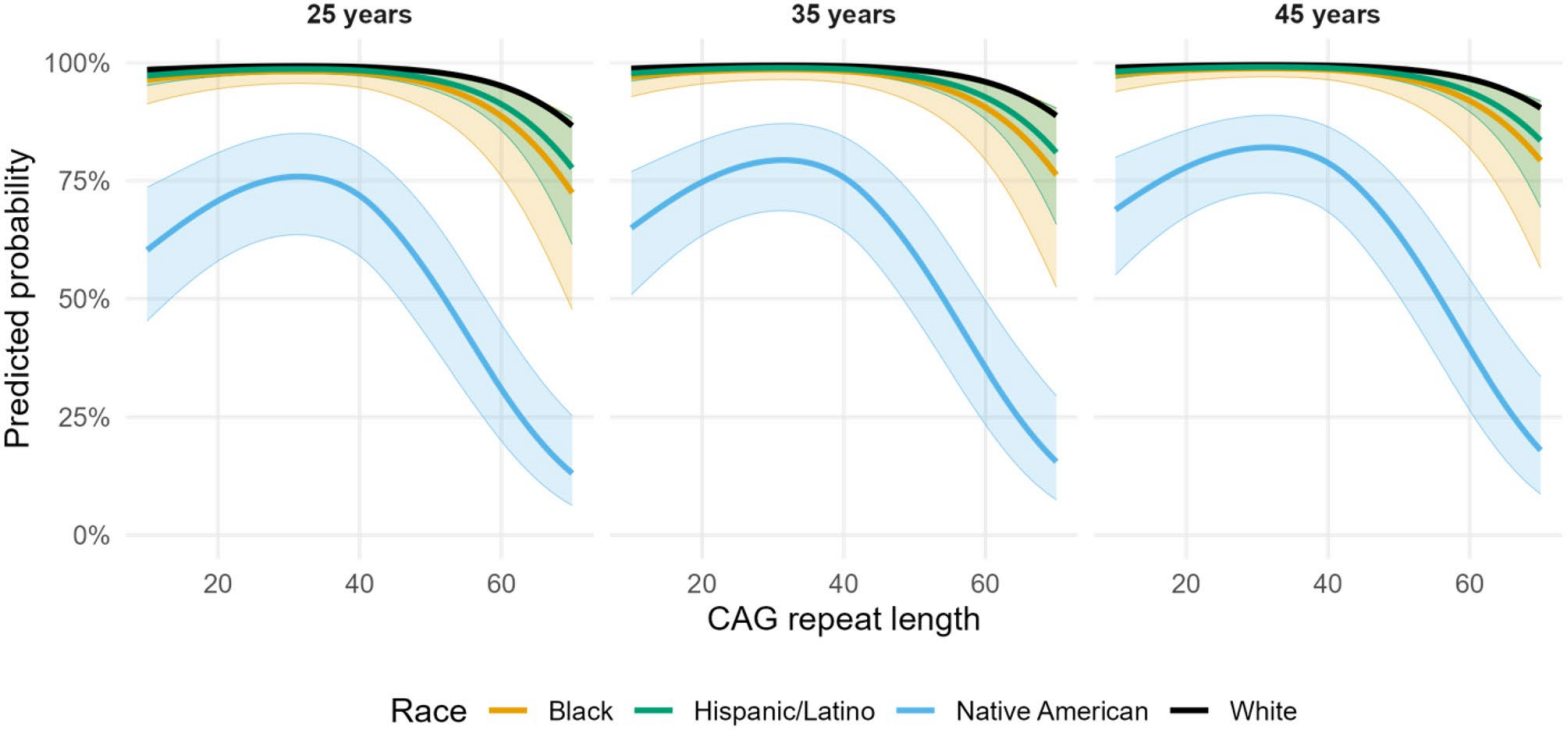
Predicted probabilities of higher educational attainment by race, parental age at symptom onset, and CAG repeat length among Enroll-HD participants (*n* = 14,392). Legend: Lines show marginal predictions from a main-effects logistic regression with 95% confidence intervals. Predictions are evaluated at age 25 years (baseline), region fixed to Northern America, averaged over the Enroll-HD sex (57% male) and affected parent (55% father) distributions. Columns indicate parental age at symptom onset (years)

The restricted cubic splines analysis signaled that a higher parental age at onset was associated with higher odds of higher educational attainment (Fig. 3, Tables A3). The relationship between CAG repeat length and higher educational attainment appeared strongly nonlinear: predicted probabilities were highest for participants with CAG repeat lengths around 30, declining for both shorter and longer repeat lengths. Figure 3 presents the predicted probability of higher educational attainment by CAG repeat length, grouped by race and stratified by parental age at onset based on the restricted cubic splines model. Predictions correspond to North American participants aged 25 years at baseline, using the average sex (57% male) and parental HD status (45% affected fathers) distributions. White, Asian, or Other participants (only White shown for reference) had higher predicted probabilities, whereas Black, Hispanic/Latino, and Native American participants had lower probabilities.

#### Sensitivity analyses

When residence was included as a covariate and cases of juvenile HD were excluded (*n* = 25), results remained consistent (Tables A4-A5). In the model considering the categorical versions of parental age at onset and CAG repeat length, residence remained statistically significant (likelihood ratio test, *p* < 0.001), with lower odds of higher educational attainment between participants residing in villages (OR = 0.73; 95% CI: 0.64–0.83) and towns (OR = 0.71; 95% CI: 0.63–0.79) when compared to cities. The full-penetrance group continued to show lower odds of higher educational attainment, though this association was no longer statistically significant (OR = 0.86; 95% CI: 0.73–1.01; *p* = 0.059). In the restricted cubic splines analysis, similar results were observed. The random-forest model identified residence as moderately important, with only a marginal improvement (AUC = 0.71; 95% CI: 0.70–0.72). Across regression models, the Hosmer-Lemeshow goodness of fit test did not provide evidence against adequate model fit.

## Discussion

To our knowledge, this is the first quantitative study that investigates how parental age at symptom onset is associated with educational outcomes in participants from the Enroll-HD study, one of the largest non-interventional studies of HD. In our explorative analysis, we found that participants whose parents had a later symptom onset were more likely to achieve higher educational attainment outcomes. Our findings are consistent with prior qualitative observations that disruption of family stability and parental support can hinder children’s educational trajectories [10].

Our explorative analysis additionally found a nonlinear association between CAG repeat length and higher educational attainment: participants with approximately 30 CAG repeats had the highest predicted probability of higher educational attainment. The inverted-U relationship is consistent with prior studies in children indicating that CAG repeat length influences neurocognitive function nonlinearly [21]. Still, these findings are associative and do not imply the use of predictive genetic testing in children, which is ethically discouraged in the absence of disease-modifying therapies.

To contextualize our findings, we compared educational attainment patterns in Enroll-HD with 2022 US Census Bureau estimates. According to national estimates, the estimated odds of higher educational attainment for Black, Hispanic/Latino, and Native American adults (aged $\:\ge\:$25 years) relative to White adults were 0.48, 0.15, and 0.49, respectively (Table A6). These US national estimates fell outside the corresponding confidence intervals for Hispanic/Latino (*n*=320) and Native American (*n* = 65) participants, with our study estimates being higher for Hispanic/Latino and lower for Native American participants. When restricting our analytical sample to participants aged $\:\ge\:$25 years in Northern America, univariate odds ratios remained similar: 0.34 (95% CI: 0.12–1.30) for Black, 0.35 (95% CI: 0.15–1.00) for Hispanic/Latino, and 0.02 (95% CI: 0.01–0.04) for Native American participants (Table A7). While Northern America includes countries beyond the US, our findings suggest relatively higher educational attainment outcomes among Hispanic/Latino participants and lower attainment among Native American participants in the Enroll-HD study.

The observed racial and ethnic disparities align with prior literature in HD and other neurodegenerative diseases such as Alzheimer’s and Parkinson’s [15, 31]. Previous studies have documented differences in time to diagnosis and disease severity across racial groups [15, 32]; for example, Black HD participants are diagnosed, on average, one year later than their White counterparts. These disparities likely reflect systemic barriers in healthcare access, early education, and socioeconomic opportunity [33]. Our results add to the racial disparity evidence by highlighting how Black, Hispanic/Latino, and Native American participants have lower odds of higher educational attainment than White participants. While our subgroup sizes were small, the magnitude of these differences underscore the need for further research that integrates race to better understand and address educational inequities in HD.

The lower odds of higher educational attainment among participants residing in Latin America compared with those in Northern America are consistent with prior reports documenting limited access to educational resources in the region [34]. Qualitative studies have described the psychological and socioeconomic toll of HD in Latin America [35], yet quantitative evidence remains scarce [31, 32]. Our results expand the evidence base by showing that participants from Latin America were approximately eight times more likely to have lower educational attainment than those from Northern America. Highlighting these regional disparities is important, as some of the highest global rates of HD occur in parts of Latin America, yet individuals from these regions remain underrepresented in HD research.

Additionally, the lower odds of higher educational attainment observed among participants from Europe and Australasia compared with those from Northern America align with national trends. According to 2022 EU estimates, the odds of attaining a higher education for those residing in the EU relative to those in the US was 0.30, whereas our study estimated an OR of 0.05 (Table A8). A similar pattern emerged for Australasia: 2024 Australia data indicate an OR of 0.27 compared with 2022 US levels, again higher than our study estimate of 0.16. While these differences appear large, our estimate reflects the broader regions and different reference years. Future studies are needed to determine whether the reduced odds of higher educational attainment observed here reflect increased disparities within the HD community or other factors, such as differences in healthcare and education access across regions.

Overall, our findings highlight the importance of family context and disease timing as potential factors for intervention. Children may experience additional obstacles suggesting a role for additional school-based accommodations, counseling services, and/or caregiver support programs tailored to families affected by early parental symptom onset. Such interventions can be coordinated among HD providers, pediatricians, social workers, and schools, do not require predictive genetic testing of children, and can be implemented based on observable parental illness. Policies that integrate educational support with health and social services for families affected by HD may therefore help reduce disparities in educational attainment while respecting ethical boundaries surrounding genetic testing in minors.

### Strengths and limitations

This study has several strengths, including a large sample, geographic diversity, and the combined use of logistic regression and machine learning to examine determinants of higher educational attainment. However, various limitations exist. First, educational attainment is a single dimension of social functioning and long-term wellbeing, and therefore does not capture the full multidimensional nature of quality of life [19]. In addition, dichotomizing educational attainment may have resulted in loss of information and obscured heterogeneity across educational systems and regions.

Second, parental age at HD onset was self-reported and is therefore subject to recall bias. Cognitive or psychiatric symptoms may precede motor symptoms by one to two decades and may not be clearly recognized at the time they occur, potentially leading to measurement error in the reported parental onset age [2]. Moreover, exclusion of participants with unobserved parental onset ages may have introduced additional selection bias. Future studies should investigate statistical methods that simultaneously account for missing and censored values of parental age at symptom onset, as treating them the same may introduce bias [36]. Third, aggregation of countries into broad geographic regions may mask differences in educational systems. Finally, while the Enroll-HD is a large multinational study, participation is voluntary thereby limiting generalizability to the broader HD population. As noted in Enroll-HD documentation, voluntary self-selection may introduce selection bias, including potential overrepresentation of individuals with better health or/and higher socioeconomic resources [26, 37]. Small sample sizes within some racial, ethnic, and regional subgroups additionally limit the precision (i.e., wide confidence intervals) and generalizability to the wider HD population.

In addition, several relevant socioeconomic variables such as household income, parental education level, type and severity of parental symptoms (e.g., motor vs. cognitive), education quality, and health insurance status were not available and could not be adjusted for. Therefore, the results presented here should be interpreted as empirical associations found, rather than causal relationships.

## Conclusion

Our findings highlight the importance of integrating parental onset, genetic-expansion, sociodemographic and regional factors into HD research. While genetic factors remain central, educational attainment–shaped by policy, environment, and family–is a modifiable determinant of long-term well-being [12, 16–19]. Recent staging systems, such as the HD Integrated Staging System [38], already recognize the role of education in disease trajectory, underscoring the need for interventions that reduce educational inequities, particularly among underrepresented racial and regional groups [32]. Our results provide novel insights into how earlier parental age at onset is associated with lower educational attainment among offspring, while also highlighting disparities across genetic-expansion, race, and regional groups. Future research should prioritize richer sociodemographic data–especially among Latin American and other underrepresented populations–to guide policies that may mitigate the burdens associated with HD and related neurodegenerative disorders.

## Supplementary Information

Below is the link to the electronic supplementary material.

Supplementary Material 1

## Data Availability

Data from the Enroll-HD study are available to qualified investigators through an application process at enroll-hd.org.

## Abbreviations

AUC: Area under the receiver operating characteristic curve
CAG: Cytosine-adenine-guanine
CI: Confidence interval
DCL: Diagnostic confidence level
HD: Huntington disease
IQR: Interquartile range
ISCED: International Standard Classification of Education
MDA: Mean Decrease in Accuracy
OR: Odds ratio
US: United States
vs.: Versus
