# Supplementary material for: Higher educational attainment in Huntington disease families: evidence from the Enroll-HD study
Source: Orphanet J Rare Dis. 2026 Apr 9;21:142. doi: 10.1186/s13023-026-04336-z (PMC13067502; doi:10.1186/s13023-026-04336-z)
Supplement: Supplementary file 1 — Supplementary Material 1 [file 13023_2026_4336_MOESM1_ESM.docx]

**Appendix to *“Higher Educational Attainment in Huntington Disease Families: Evidence from the Enroll-HD Study”***

**Authors:** Jesus E. Vazquez^1^, Dewei Lin^2^, Adys Mendizabal^3^, Amy C. Ogilvie^4^, Elizabeth Stuart^1^, Tanya P. Garcia^2^

**Institutions and affiliations:**

^1^Department of Biostatistics, Bloomberg School of Public Health, Johns Hopkins University, Baltimore, MD, United States

^2^Department of Biostatistics, Gillings School of Global Public Health, The University of North Carolina at Chapel Hill, Chapel Hill, NC, United States

^3^Department of Neurology, University of California, Los Angeles, CA, United States

^4^Department of Neurology, Health Services Research Division, Ohio State University, Columbus, OH, United States

**Jesus E. Vazquez (Corresponding author)**

Department of Biostatistics

Johns Hopkins University

615 N Wolfe Street, Office E3139

Baltimore, MD 21205

Email: jesusepfvazquez@gmail.com

Phone: +1 410-955-3067

| Page | Table of Contents | |
| --- | --- | --- |
|  |  |  |
| 2 |  | **Table A1:** Logistic regression odds ratio estimates of higher educational attainment, Enroll-HD (n=14,392) |
| 3 |  | **Table A2:** Univariate odds ratio estimates of higher educational attainment by parental age at onset and region, Enroll-HD |
| 4 |  | **Table A3:** Restricted cubic splines logistic regression log-odds ratio estimates of higher educational attainment, Enroll-HD (n=14,392) |
| 5 |  | **Table A4:** Logistic regression odds ratio estimates of higher educational attainment adjusted for residence, Enroll-HD (n=14,367) |
| 6 |  | **Table A5:** Restricted cubic splines logistic regression log-odds ratio estimates of higher educational attainment adjusted for residence, Enroll-HD (n=14,367) |
| 7 |  | **Table A6:** Univariate odds ratio estimates of higher educational attainment among adults aged $\geq25$ in Northern America, Enroll-HD (n=4,411) |
| 8 |  | **Table A7:** Univariate odds ratio estimates of higher educational attainment by race, United States (2022) |
| 9 |  | **Table A8:** Univariate odds ratio estimates of higher educational attainment by country (2022) |
|  |  |  |
| 11 | **References** | |

**Table A1:** Logistic regression odds ratio estimates of higher educational attainment, Enroll-HD (n=14,392)

|  | Odds ratio | 95% CI | | $\boldsymbol{p}$-value | Importance$\boldsymbol{}^{\boldsymbol{a}}$ |
| --- | --- | --- | --- | --- | --- |
| Age (5 years)$\boldsymbol{}^{\boldsymbol{b}}$ | 0.868 | 0.850 | 0.885 | <0.001 | High |
| Parental age at onset group |  |  |  |  | Moderate |
| 25–34 | 1.276 | 0.827 | 1.968 | 0.270 |  |
| 35–44 | 1.646 | 1.081 | 2.508 | 0.020 |  |
| $\boldsymbol{\geq}$45 | 2.347 | 1.542 | 3.572 | <0.001 |  |
| $\boldsymbol{<}$25 | Ref |  |  |  |  |
| Parent affected (Father) | 0.982 | 0.893 | 1.080 | 0.720 | Low |
| CAG repeat length group |  |  |  |  | Moderate |
| Intermediate (27–35) | 0.897 | 0.512 | 1.573 | 0.705 |  |
| Reduced penetrance (36–39) | 1.006 | 0.749 | 1.349 | 0.970 |  |
| Full penetrance (40–59) | 0.836 | 0.711 | 0.982 | 0.029 |  |
| High ($\boldsymbol{\geq}$60) | 0.174 | 0.104 | 0.289 | <0.001 |  |
| Normal | Ref |  |  |  |  |
| Sex (Female) | 1.046 | 0.952 | 1.149 | 0.350 | Low |
| Region |  |  |  |  | High |
| Europe | 0.055 | 0.044 | 0.068 | <0.001 |  |
| Australasia | 0.163 | 0.111 | 0.243 | <0.001 |  |
| Latin America | 0.114 | 0.064 | 0.204 | <0.001 |  |
| Northern America | Ref |  |  |  |  |
| Race |  |  |  |  | High |
| Black | 0.378 | 0.156 | 0.917 | 0.031 |  |
| Hispanic/Latino | 0.547 | 0.349 | 0.860 | 0.009 |  |
| Other | 2.671 | 1.548 | 4.800 | <0.001 |  |
| Native American | 0.023 | 0.013 | 0.041 | <0.001 |  |
| Asian | 0.972 | 0.551 | 1.719 | 0.920 |  |
| White | Ref |  |  |  |  |
| Note: Abbreviations: CI (Confidence interval), Ref (Reference), MDA (Mean Decrease in Accuracy).  $\boldsymbol{}^{\boldsymbol{a}}$ Importance of the variable was measured using MDA from a random forest model (1,000 trees; 7 predictors; Area under curve = 0.705 with 95% CI 0.696-0.716). Larger MDA values indicate greater predictive relevance and were operationalized as high (MDA $\boldsymbol{>}$25), moderate (10–25), or low ($\boldsymbol{<}$10).  $\boldsymbol{}^{\boldsymbol{b}}$ Age scaled at 5-years of age. | | | | | |

**Table A2:** Univariate odds ratio estimates of higher educational attainment by parental age at onset and region, Enroll-HD

|  | **n** | **Odds ratio (95% CI)** | $\boldsymbol{p}$-value$\boldsymbol{}^{\boldsymbol{a}}$ |
| --- | --- | --- | --- |
|  |  |  |  |
| Europe (n = 8,875) |  |  |  |
| Parental age of onset group |  |  |  |
| 35–44 | 2,361 | 1·157 (0·967, 1·381) | 0·105 |
| $\boldsymbol{\geq}$45 | 5,643 | 1·325 (1·124, 1·558) | 0·001 |
| $\boldsymbol{<35}$ | 871 | Ref |  |
|  |  |  |  |
| Australasia (n = 525) |  |  |  |
| Parental age of onset group |  |  |  |
| 35–44 | 119 | 0·645 (0·065, 3·318) | 0·730 |
| $\boldsymbol{\geq}$45 | 366 | 0·569 (0·064, 2·394) | 0·760 |
| $\boldsymbol{<35}$ | 40 | Ref |  |
|  |  |  |  |
| Latin America (n = 150) |  |  |  |
| Parental age of onset group |  |  |  |
| 35–44 | 43 | 2·865 (0·562, 14·753) | 0·140 |
| $\boldsymbol{\geq}$45 | 89 | 1·511 (0·373, 5·305) | 0·530 |
| $\boldsymbol{<35}$ | 18 | Ref |  |
|  |  |  |  |
| Northern America (n = 4,842) |  |  |  |
| Parental age of onset group |  |  |  |
| 35–44 | 1,310 | 1·606 (0·912, 2·790) | 0·088 |
| $\boldsymbol{\geq}$45 | 2,940 | 2·356 (1·392, 3·888) | 0·001 |
| $\boldsymbol{<35}$ | 592 | Ref |  |
|  |  |  |  |
| Note: Abbreviations: Ref (Reference). Higher educational attainment operationalized as ISCED$\boldsymbol{\geq3}$.  $\boldsymbol{}^{\boldsymbol{a}}$ $\boldsymbol{p}$-values were obtained using the Fisher’s exact test, which evaluates the null hypothesis of an odds ratio equal to 1 using the hypergeometric distribution (fixed columns and row totals). | | | |

**Table 2A Caption:** Because educational systems may differ across regions, we additionally conducted a stratified univariate analysis of the association between parental age at onset and higher educational attainment. Given the small counts of offspring whose parents began experiencing symptoms before age 25 in Australasia and Latin America, we grouped these with the 25-34 category to form a combined <35 group. Among those from Europe, parental onset between ages 35-44 and ≥45 was associated with ORs of 1.16 (95% CI: 0.97-1.38) and 1.33 (95% CI: 1.12-1.56), respectively, when compared with <35. Similarly, for those residing in Northern America, parental onset between ages 35-44 (OR=1.61, 95% CI: 0.91-2.79) and ≥45 (OR=2.36, 95% CI: 1.39-3.89) was associated with increased odds of higher educational attainment. Together, these findings further support that later parental age at symptom onset is associated with higher educational in offspring.

**Table A3:** Restricted cubic spline logistic regression log-odds ratio estimates of higher educational attainment, Enroll-HD (n=14,392)

|  | Estimate | 95% CI | | $\boldsymbol{p}$-value |
| --- | --- | --- | --- | --- |
| Intercept | 3.098 | 2.337 | 3.859 | <0.001 |
| Parent age at onset (spline terms) $\boldsymbol{}^{\boldsymbol{a}}$ |  |  |  |  |
| 1st spline term | 0.834 | 0.455 | 1.213 | <0.001 |
| 2nd spline term | 1.423 | -0.058 | 2.904 | 0.060 |
| 3rd spline term | 0.352 | -0.265 | 0.968 | 0.260 |
| Age (5 years)$\boldsymbol{}^{\boldsymbol{a}}$ | -0.175 | -0.198 | -0.153 | <0.001 |
| Parent affected (Father) | 0.025 | -0.071 | 0.122 | 0.605 |
| CAG repeat length (spline terms) |  |  |  |  |
| 1st spline term | -0.799 | -1.123 | -0.475 | <0.001 |
| 2nd spline term | -0.553 | -1.335 | 0.229 | 0.170 |
| 3rd spline term | -2.879 | -3.531 | -2.226 | <0.001 |
| Sex (Female) | 0.037 | -0.058 | 0.132 | 0.450 |
| Region |  |  |  |  |
| Europe | -2.896 | -3.120 | -2.673 | <0.001 |
| Australasia | -1.824 | -2.216 | -1.433 | <0.001 |
| Latin America | -2.126 | -2.707 | -1.545 | <0.001 |
| Northern America | Ref |  |  |  |
| Race |  |  |  |  |
| Black | -0.901 | -1.797 | -0.005 | 0.049 |
| Hispanic/Latino | -0.620 | -1.071 | -0.168 | 0.007 |
| Other | 1.023 | 0.476 | 1.571 | <0.001 |
| Native American | -3.758 | -4.320 | -3.196 | <0.001 |
| Asian | -0.041 | -0.611 | 0.530 | 0.890 |
| White | Ref |  |  |  |
| Note: Abbreviations: CI (Confidence interval), Ref (Reference).  $\boldsymbol{}^{\boldsymbol{a}}$ Age centered at 45 years and scaled by 5. Parental age at onset (centered at 45) and CAG repeat length were modeled using restricted cubic splines (3 knots). | | | | |

##

**Table A4:** Logistic regression odds ratio estimates of higher educational attainment adjusted for residence, Enroll-HD (n=14,367)

|  | Odds ratio | 95% CI | | $\boldsymbol{p}$-value | Importance$\boldsymbol{}^{\boldsymbol{a}}$ |
| --- | --- | --- | --- | --- | --- |
| Age (5 years)$\boldsymbol{}^{\boldsymbol{b}}$ | 0.869 | 0.852 | 0.886 | <0.001 | High |
| Parental age at onset group |  |  |  |  | Moderate |
| 25–34 | 1.323 | 0.853 | 2.052 | 0.210 |  |
| 35–44 | 1.686 | 1.103 | 2.578 | 0.016 |  |
| $\boldsymbol{\geq}$45 | 2.405 | 1.574 | 3.675 | <0.001 |  |
| $\boldsymbol{<}$25 | Ref |  |  |  |  |
| Parent affected (Father) | 0.984 | 0.894 | 1.082 | 0.740 | Low |
| CAG repeat length group |  |  |  |  | Moderate |
| Intermediate (27–35) | 0.874 | 0.498 | 1.535 | 0.640 |  |
| Reduced penetrance (36–39) | 1.037 | 0.772 | 1.392 | 0.810 |  |
| Full penetrance (40–59) | 0.856 | 0.728 | 1.006 | 0.059 |  |
| High ($\boldsymbol{\geq}$60) | 0.188 | 0.107 | 0.331 | <0.001 |  |
| Normal | Ref |  |  |  |  |
| Sex (Female) | 1.045 | 0.950 | 1.149 | 0.360 | Low |
| Region |  |  |  |  | High |
| Europe | 0.058 | 0.047 | 0.073 | <0.001 |  |
| Australasia | 0.169 | 0.114 | 0.250 | <0.001 |  |
| Latin America | 0.107 | 0.059 | 0.191 | <0.001 |  |
| Northern America | Ref |  |  |  |  |
| Residence |  |  |  |  | Moderate |
| Rural | 0.830 | 0.667 | 1.034 | 0.097 |  |
| Village | 0.729 | 0.638 | 0.832 | <0.001 |  |
| Town | 0.709 | 0.634 | 0.794 | <0.001 |  |
| City | Ref |  |  |  |  |
| Race |  |  |  |  | Moderate |
| Black | 0.346 | 0.142 | 0.841 | 0.019 |  |
| Hispanic/Latino | 0.509 | 0.323 | 0.803 | 0.004 |  |
| Other | 2.515 | 1.457 | 4.339 | <0.001 |  |
| Native American | 0.024 | 0.014 | 0.042 | <0.001 |  |
| Asian | 0.919 | 0.520 | 1.627 | 0.770 |  |
| White | Ref |  |  |  |  |
| Note: Abbreviations: CI (Confidence interval), Ref (Reference), AUC (Area under curve), MDA (Mean Decrease in Accuracy). Sensitivity analysis excludes $\boldsymbol{n=25}$ participants with juvenile HD (defined as DCL=4 before age 20).  $\boldsymbol{}^{\boldsymbol{a}}$ Importance measured via MDA (random forest; 600 trees; 8 predictors). High $\boldsymbol{>}$ 25, Moderate 10–25, Low $\boldsymbol{<}$ 10.  $\boldsymbol{}^{\boldsymbol{b}}$ Age (per 5-year increase). | | | | | |

**Table A5:** Restricted cubic splines logistic regression log-odds ratio estimates of higher educational attainment adjusted for residence, Enroll-HD (n=14,367)

|  | Estimate | 95% CI | | $\boldsymbol{p}$-value |
| --- | --- | --- | --- | --- |
| Intercept | 3.221 | 2.453 | 3.989 | <0.001 |
| Parent age at onset (spline terms) $\boldsymbol{}^{\boldsymbol{a}}$ |  |  |  |  |
| 1st spline term | 0.845 | 0.465 | 1.226 | <0.001 |
| 2nd spline term | 1.431 | -0.058 | 2.921 | 0.060 |
| 3rd spline term | 0.335 | -0.285 | 0.954 | 0.290 |
| Age (5 years)$\boldsymbol{}^{\boldsymbol{a}}$ | -0.173 | -0.196 | -0.151 | <0.001 |
| Parent affected (Father) | 0.026 | -0.070 | 0.123 | 0.600 |
| CAG repeat length (spline terms) |  |  |  |  |
| 1st spline term | -0.745 | -1.075 | -0.414 | <0.001 |
| 2nd spline term | -0.570 | -1.390 | 0.251 | 0.170 |
| 3rd spline term | -2.909 | -3.636 | -2.181 | <0.001 |
| Sex (Female) | 0.036 | -0.059 | 0.131 | 0.460 |
| Region |  |  |  |  |
| Europe | -2.836 | -3.060 | -2.612 | <0.001 |
| Australasia | -1.791 | -2.183 | -1.399 | <0.001 |
| Latin America | -2.187 | -2.771 | -1.603 | <0.001 |
| Northern America | Ref |  |  |  |
| Residence |  |  |  |  |
| Rural | -0.174 | -0.394 | 0.047 | 0.122 |
| Village | -0.295 | -0.428 | -0.162 | <0.001 |
| Town | -0.330 | -0.443 | -0.217 | <0.001 |
| City | Ref |  |  |  |
| Race |  |  |  |  |
| Black | -0.977 | -1.876 | -0.078 | 0.033 |
| Hispanic/Latino | -0.688 | -1.143 | -0.234 | 0.003 |
| Other | 0.972 | 0.423 | 1.520 | <0.001 |
| Native American | -3.740 | -4.305 | -3.174 | <0.001 |
| Asian | -0.095 | -0.668 | 0.478 | 0.750 |
| White | Ref |  |  |  |
| Note: Abbreviations: CI (Confidence interval), Ref (Reference). Sensitivity analysis excludes $\boldsymbol{n=25}$ participants with juvenile HD (defined as DCL=4 before age 20).  $\boldsymbol{}^{\boldsymbol{a}}$ Age centered at 45 years and scaled by 5. Parental age at onset (centered at 45) and CAG repeat length were modeled using restricted cubic splines (3 knots). | | | | |

**Table A6:** Univariate odds ratio estimates of higher educational attainment, United States (2022)

|  | Percentage with higher educational attainment$\boldsymbol{}^{\boldsymbol{a}}$ | Odds ratio$\boldsymbol{}^{\boldsymbol{b}}$ |
| --- | --- | --- |
| Race |  |  |
| Black | 90.5% | 0.153 |
| Hispanic/Latino | 75.2% | 0.480 |
| Native American | 89.9% | 0.449 |
| White | 95.2% | Ref |
| Note: Abbreviations: Ref (Reference). Data available in United States Census Bureau^1^. Data are presented as percentages as raw sample sizes were not provided by the data sources.  $\boldsymbol{}^{\boldsymbol{a}}$ Percent of population aged 25 and older, higher educational attainment operationalized as ISCED$\boldsymbol{\geq}\boldsymbol{3}$.  $\boldsymbol{}^{\boldsymbol{b}}$ Odds ratio is calculated as the ratio of odds of higher educational attainment for each group: $\text{OR}\boldsymbol{=}\frac{\boldsymbol{p}_{\boldsymbol{1}}\boldsymbol{/(}\boldsymbol{1}\boldsymbol{-}\boldsymbol{p}_{\boldsymbol{1}}\boldsymbol{)}}{\boldsymbol{p}_{\boldsymbol{2}}\boldsymbol{/(}\boldsymbol{1}\boldsymbol{-}\boldsymbol{p}_{\boldsymbol{2}}\boldsymbol{)}}$, where $\boldsymbol{p}_{\boldsymbol{1}}$ is the group’s proportion and $\boldsymbol{p}_{\boldsymbol{2}}$ is the reference group’s proportion. | | |

**Table A7:** Univariate odds ratio estimates of higher educational attainment among adults aged $\geq25$ in Northern America, Enroll-HD (n=4,411)

|  | **n** | **Odds ratio (95% CI)** | $\boldsymbol{p}$-value$\boldsymbol{}^{\boldsymbol{a}}$ |  |
| --- | --- | --- | --- | --- |
| Race |  |  |  |  |
| Black | 95 | 0.337 (0.121, 1.304) | 0.055 |  |
| Hispanic/Latino | 146 | 0.346 (0.146, 0.995) | 0.023 |  |
| Native American | 57 | 0.022 (0.012, 0.042) | <0.0001 |  |
| White | 4,113 | Ref |  |  |
| Note: Abbreviations: CI (Confidence interval), Ref (Reference). Higher educational attainment operationalized as ISCED$\boldsymbol{\geq3}$.  $\boldsymbol{}^{\boldsymbol{a}}$ $\boldsymbol{p}$-values were obtained using Fisher’s exact test, which evaluates the null hypothesis of an odds ratio equal to 1 using hypergeometric distribution. | | | | |

**Table A8:** Univariate odds ratio estimates of higher educational attainment by country (2022)

|  | Percent with higher educational attainment$\boldsymbol{}^{\boldsymbol{a}}$ | Odds ratio$\boldsymbol{}^{\boldsymbol{b}}$ |
| --- | --- | --- |
| Country/region |  |  |
| Europe$\boldsymbol{}^{\boldsymbol{c}}$ | 75.2% | 0.297 |
| Australia$\boldsymbol{}^{\boldsymbol{d}}$ | 73.1% | 0.612 |
| United States$\boldsymbol{}^{\boldsymbol{e}}$ | 91.2% | Ref |
| Note: Abbreviations: Ref (Reference). Data are presented as percentages as raw sample sizes were not provided by the data sources.  $\boldsymbol{}^{\boldsymbol{a}}$ Percent of population aged 25 and older, higher educational attainment operationalized as ISCED$\boldsymbol{\geq}\boldsymbol{3}$.  $\boldsymbol{}^{\boldsymbol{b}}$ Odds ratio is calculated as the ratio of odds of higher educational attainment for each group: $\text{OR}\boldsymbol{=}\frac{\boldsymbol{p}_{\boldsymbol{1}}\boldsymbol{/(}\boldsymbol{1}\boldsymbol{-}\boldsymbol{p}_{\boldsymbol{1}}\boldsymbol{)}}{\boldsymbol{p}_{\boldsymbol{2}}\boldsymbol{/(}\boldsymbol{1}\boldsymbol{-}\boldsymbol{p}_{\boldsymbol{2}}\boldsymbol{)}}$, where $\boldsymbol{p}_{\boldsymbol{1}}$ is the group’s proportion and $\boldsymbol{p}_{\boldsymbol{2}}$ is the reference group’s proportion.  $\boldsymbol{}^{\boldsymbol{c}}$ Data available in European Statistical Office (2022)^2^.  $\boldsymbol{}^{\boldsymbol{d}}$ Data available in Australian Bureau of Statistics (2022)^3^.  $\boldsymbol{}^{\boldsymbol{e}}$ Data available in United States Census Bureau (2022)^1^. | | |

**References**

1. Bureau USC. Census Bureau Releases New Educational Attainment Data. 2023. <https://www.census.gov/newsroom/press-releases/2023/educational-attainment-data.html> (accessed 19 November 2025 2025).

2. Eurostat. Educational attainment. 2025. <https://ec.europa.eu/eurostat/statistics-explained/index.php?title=Educational_attainment_statistics> (accessed 19 November 2025 2025).

3. Statistics ABo. Education attainment. 2025. <https://www.abs.gov.au/statistics/measuring-what-matters/measuring-what-matters-themes-and-indicators/prosperous/education-attainment#metrics> (accessed 19 November 2025.
